# Understanding and Exploiting the Effect of Tuberculosis Antimicrobials on Host Mitochondrial Function and Bioenergetics

**DOI:** 10.3389/fcimb.2020.00493

**Published:** 2020-09-15

**Authors:** Christina Cahill, James Joseph Phelan, Joseph Keane

**Affiliations:** TB Immunology Group, Department of Clinical Medicine, Trinity Translational Medicine Institute, Trinity College Dublin, Dublin, Ireland

**Keywords:** antimicrobials, tuberculosis, mitochondrial function, bioenergetics, oxidative phosphorylation, glycolysis, cellular metabolism

## Abstract

Almost 140 years after its discovery, tuberculosis remains the leading infectious cause of death globally. For half a century, patients with drug-sensitive and drug-resistant tuberculosis have undergone long, arduous, and complex treatment processes with several antimicrobials that primarily function through direct bactericidal activity. Long-term utilization of these antimicrobials has been well-characterized and associated with numerous toxic side-effects. With the prevalence of drug-resistant strains on the rise and new therapies for tuberculosis urgently required, a more thorough understanding of these antimicrobials is a necessity. In order to progress from the “one size fits all” treatment approach, understanding how these antimicrobials affect mitochondrial function and bioenergetics may provide further insight into how these drugs affect the overall functions of host immune cells during tuberculosis infection. Such insights may help to inform future studies, instigate discussion, and help toward establishing personalized approaches to using such antimicrobials which could help to pave the way for more tailored treatment regimens. While recent research has highlighted the important role mitochondria and bioenergetics play in infected host cells, only a small number of studies have examined how these antimicrobials affect mitochondrial function and immunometabolic processes within these immune cells. This short review highlights how these antimicrobials affect key elements of mitochondrial function, leading to further discussion on how they affect bioenergetic processes, such as glycolysis and oxidative phosphorylation, and how antimicrobial-induced alterations in these processes can be linked to downstream changes in inflammation, autophagy, and altered bactericidal activity.

## Introduction

Tuberculosis (TB) is caused by infection with *Mycobacterium tuberculosis (Mtb)* and remains the leading cause of death by a single infectious agent in the world. In 2018, TB disease resulted in ~1.5 million deaths and still presents a major clinical challenge globally (WHO, 2019a). Treatment for patients with drug-sensitive TB involves a long, grueling, and complex drug regimen, often involving the administration of the first line antimicrobials rifampicin, isoniazid, pyrazinamide, and ethambutol. Poor compliance and inadequate treatment have led to the emergence of drug-resistant strains, resulting in multi-drug-resistant TB (MDR-TB) and extensively drug-resistant TB (XDR-TB) (Kliiman and Altraja, 2009; Liang et al., 2012). The regimen to tackle these drug-resistant strains further complicates treatment, increases treatment length and requires the management of multiple toxic drugs (Srivastava et al., 2011). Treatment of drug-resistant TB can be patient-specific, based on which drugs the bacteria are specifically susceptible to, which often includes multiple second line drugs. Second line TB antimicrobials are categorized by the WHO into three groups, which are ranked in order of priority (Table 1). Table 1 also summarizes which antimicrobials will be discussed and lists those with unknown effects on mitochondria and bioenergetics in host cells. Treatment of MDR-TB is recommended to include all three Group A agents and at least one Group B agent. TB strains which are resistant to Group A and B antimicrobials, such as in XDR-TB, are treated with the addition of one or more Group C agents (WHO, 2019b).

**Table 1:** Grouping of medicines recommended for use in longer MDR-TB regimens.

**Group**	**Antimicrobial**
**Group A** Include all three medicines	Moxifloxacin (MXF)*/Levofloxacin (LFX)*
	Bedaquiline (BDQ)*
	Linezolid (LZD)*
**Group B** Add one or both medicines	Clofazimine (CFZ)*
	Cycloserine^#^/Terizidone^#^
**Group C** Add to complete the regimen/when medicines from Group A and B cannot be used	Ethambutol (EMB)*
	Delamanid^#^
	Pyrazinamide (PZA)*
	Imipenem–cilastatin^#^/Meropenem^#^
	Amikacin^#^/Streptomycin^#^
	Ethionamide^#^/Prothionamide^#^
	P-aminosalicylic acid^#^

*Table adapted from the WHO resource (WHO, 2019b)*.

* *Indicates antimicrobials discussed in the current review*.

# *Indicates antimicrobials with unknown effects on mitochondria and bioenergetics of host cells*.

**Figure 1:**
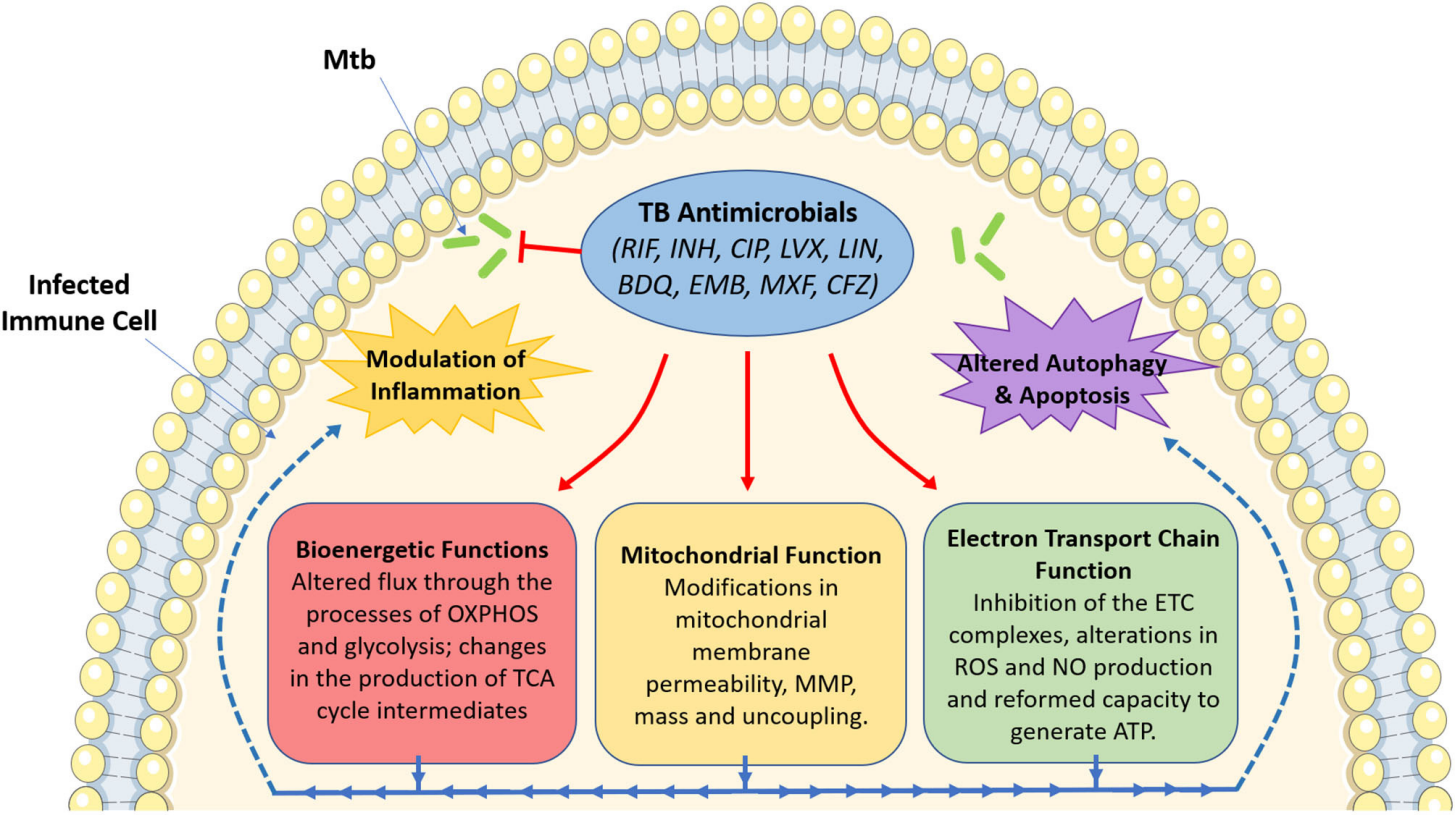
An overview of the effect of tuberculosis antimicrobials on host mitochondrial function, bioenergetics and immune cell function. Tuberculosis (TB) antimicrobials, that are routinely used to fight TB infection, could affect a range of cellular processes, particularly mitochondrial function and various bioenergetic processes. These antimicrobials could alter MMP (isoniazid and ciprofloxacin), mitochondrial membrane permeability (rifampicin, ciprofloxacin and levofloxacin), mitochondrial mass (linezolid), or result in mitochondrial uncoupling (ciprofloxacin and levofloxacin). They could also inhibit electron transport chain complex activity (ciprofloxacin, levofloxacin, linezolid, bedaquiline, ethambutol, and rifampicin), ROS production (rifampicin and isoniazid), NO production (ciprofloxacin and levofloxacin), or ATP production (rifampicin, isoniazid and ciprofloxacin). Moreover, these antimicrobials have been shown to alter metabolic flux through OXPHOS (rifampicin, linezolid, bedaquiline, and clofazimine), glycolysis (bedaquiline, rifampicin, linezolid, moxifloxacin, ciprofloxacin, and clofazimine), while also affecting the production of TCA cycle intermediates (clofazimine). Such changes in mitochondrial function and host bioenergetics could also be linked with alterations in apoptosis (levofloxacin, ciprofloxacin, and linezolid), the autophagic process (isoniazid and bedaquiline) and a variety of inflammatory phenotypes (rifampicin, ciprofloxacin, levofloxacin, linezolid, bedaquiline, moxifloxacin, and clofazimine). Image produced with the aid of Servier Medical Art software (see copyright license at https://smart.servier.com).

The use of these antimicrobials has been the mainstay for TB treatment for almost half a century now. Despite the multitude of readily available antimicrobials, TB still persists as a major global infectious hazard due to the increasing prevalence of drug-resistant strains, as well as other factors such as immunosuppression, the prevalence of latent TB infection and its mode of transmission. This necessitates the need to identify better therapies to help simplify and shorten treatment times. Recently, much attention has turned to the use of host-directed therapy (HDT) to help improve TB treatment through the enhancement of immune responses in infected host immune cells (Kaufmann et al., 2018). Such HDT strategies have been hypothesized to work through boosting anti-inflammatory (Kaufmann et al., 2018), pro-inflammatory (Dawson et al., 2009), and immunometabolic processes (Phelan et al., 2020). Interestingly, in addition to their bactericidal effects, evidence suggests that TB antimicrobials could also help during TB disease through the modulation of host immune responses through host-directed strategies. Such approaches include altering mitochondrial function, metabolism, cell death pathways, autophagy, and chemokine and cytokine production in immune cells infected with *Mtb*. Although many TB antimicrobials have yet to be investigated in terms of host immune modulation, this review aims to demonstrate the importance of such future studies. As Figure 1 illustrates, this brief review outlines the potential importance of studying these host effects, specifically focusing on how these antimicrobials could alter mitochondrial function and cellular bioenergetics, effects which may prove to be both detrimental and/or beneficial during standard TB treatment.

## Tuberculosis Antimicrobials Alter Optimal Mitochondrial Function and Dynamics

Central to the immunometabolic demands of host immune cells are the mitochondria. *Mtb* has adapted to escape immune responses through the utilization of several immunological devices, including targeting cellular organelles such as the mitochondria. In fact, mitochondrial function and morphology are altered as a result of *Mtb* infection, including mitochondrial size, shape, number, distribution, and fragmentation (Fine-Coulson et al., 2015). Infection of human monocyte-derived macrophages (hMDMs) with *Mtb* H37Rv also results in the release of cytochrome c from the mitochondria and a depletion of mitochondrial membrane potential (MMP) which profoundly changes how these cells respond to *Mtb* infection (Chen et al., 2006). Optimal mitochondrial function contributes to the production of ATP and reactive oxygen species (ROS), and to the regulation of cell death pathways. Thus, investigating how specific TB antimicrobials could alter these processes could lead to novel HDT approaches for TB. Conversely, identifying and understanding how these antimicrobials illicit unwanted effects in the host would also be advantageous.

The mitochondria play key protective roles during TB infection (Abarca-Rojano et al., 2003; Dubey, 2016). Indeed, various antimicrobials have been shown to mechanistically alter mitochondrial function. As will be discussed, TB antimicrobials predominantly affect mitochondrial function through the modulation of mitochondrial mass, MMP and ROS production, and by affecting the expression and function of specific complexes on the electron transport chain. For example, rifampicin (RIF) has long been associated with alterations in mitochondrial function since the 1970s, when it was discovered to inhibit mitochondrial RNA polymerase and RNA synthesis in rat liver (Gadaleta et al., 1970; Buss et al., 1978). Release of cytochrome c from the mitochondria is also observed upon RIF treatment of the kidney epithelial SPEV cell line, indicating a change in mitochondrial membrane permeability and an induction of the apoptotic pathways (Erokhina et al., 2013). RIF also decreases the activity of catalase, glutathione peroxidase, and superoxide dismutase in rat liver mitochondria (Rana et al., 2006). These enzymes are essential for the removal of excess mitochondrial ROS, which is, unsurprisingly, increased in RIF-treated SPEV cell lines (Erokhina et al., 2013). These mitochondrial alterations induced by RIF are likely to be a cause of hepatoxicity and the rarer acute liver toxicity in RIF-treated patients (Kora et al., 2018). Although associated with mitochondrial damage, ROS is also required for inflammasome function and for the processing of pro-IL-1β (Gleeson et al., 2016). In fact, RIF-treated HepG2 hepatocytes display an upregulation in IL-1β, IL-8, and IP-10 expression (Yuhas et al., 2011). In contrast to this however, RIF was found to decrease IL-1β and TNF-α production but increase IL-6 and IL-10 production in LPS and *Staphylococcus aureus* stimulated monocytes (Ziglam et al., 2004), also suggesting an anti-inflammatory role for this antibiotic. A protective role for RIF has also been hypothesized. In mice liver samples RIF is shown to activate AMPKα-mediated Nrf2 translocation to the nucleus, which regulates antioxidant expression, thus reducing ROS production, alleviating mitochondrial stress, and preventing apoptosis and inflammation (Lee et al., 2020). Notably, the differences in mitochondrial function and bioenergetics observed in LPS-stimulated cells and those infected with *S. aureus* and how these findings translate to the TB setting should also be carefully considered as different infectious models likely modulate downstream processes differently depending on the pattern recognition receptors, the pattern associated molecular patterns (PAMPs) and the damage associated molecular patterns (DAMPs) that are engaged.

Treatment with the first line drug pyrazinamide (PZA) increases ROS production and decreases expression of liver fatty acid binding protein (L-FABP) as well as its target gene, PPAR-α, in zebrafish larvae (Zhang et al., 2016). Although this is accompanied by increased oxidative stress and the expression of TNF-α and TGF-β, future avenues of research are required to explore if some of these PZA-induced effects are also mediated through the mitochondria (Zhang et al., 2016). Another first line antimicrobial isoniazid (INH), which acts by inhibiting the synthesis of an essential component of the cell wall in *Mtb*, mycolic acid (Winder and Collins, 1970), increases ROS production and lipid peroxidation and reduces MMP in isolated rat liver mitochondria, as measured by flow cytometric and malonedialdehyde assays respectively (Ahadpour et al., 2016). Moreover, in rat brain mitochondria, INH decreases glutathione content and ATP production. These INH-induced effects in rat brain and liver mitochondria resulted from reduced activity of mitochondrial complexes I and III. However, INH also reduced complex II activity in rat liver mitochondria (Ahadpour et al., 2016). Despite these impairments in mitochondrial complex function, ROS production, induced by INH, is also required for the initiation and activation of autophagy and microbial clearance in *Mtb* infected bone marrow-derived macrophages (BMDMs) (Kim et al., 2012). Therefore, although responsible for defects in mitochondrial function, INH also has the potential to help aid in *Mtb* clearance by promoting the autophagic process, further highlighting its ability to modulate host function and augment its therapeutic potential.

The second line fluoroquinolones ciprofloxacin (CIP) and levofloxacin (LVX) inhibit *Mtb* cell division during infection (Pan and Fisher, 1997). While CIP is not recommended by the WHO for TB treatment as it is linked with increased resistance (Gumbo et al., 2005), ~30% of patients in high TB burden areas still receive CIP for TB treatment (Wells et al., 2011). CIP, which inhibits mycobacterial topoisomerase IV, can also affect mammalian topoisomerase II, in particular its mitochondrial isoform thereby disturbing mitochondrial DNA (mtDNA) replication which is integral for optimal mitochondrial function. Indeed, in CIP-treated human lymphoblastoid T cells, this mtDNA disturbance reduces oxygen consumption and cellular capacity to produce ATP. CIP also inhibits Ca^2+^ entry and decreases MMP in these cells (Kozieł et al., 2006). Importantly, LVX and CIP also increase nitric oxide (NO) production in these tissues, a product widely known to interfere with various elements of mitochondrial function such as the abrogation of cytochrome c oxidase activity, inhibition of the electron transport chain (ETC), mitochondrial uncoupling, mitochondrial permeability, and mitochondrial-induced cell death (Poderoso et al., 2019). NO has been implicated as an inflammatory agent (Barnes and Liew, 1995) but may also have anti-inflammatory effects (Granger and Kubes, 1996). In fact, CIP and LVX were found to elicit differential effects in LPS-stimulated mice; both CIP and LVX decreased serum IL-1β levels. However, CIP treatment decreased TNF-α but increased IL-6 levels in the serum (Ogino et al., 2009). Even though these studies highlight the potential immunomodulatory effects that these antimicrobials can confer on different cellular process, the toxic side effects of tuberculosis antimicrobials must also be considered.

The ribosomal inhibitor, linezolid (LZD), elicits a range of toxic side effects after long-term treatment, with disturbances in mitochondrial function thought to be the main cause of the majority of these adverse effects (Soriano et al., 2005; Santini et al., 2017). LZD decreases protein levels and the enzymatic activity of various ETC enzymes including complexes I, III, IV, and V in rat muscle and liver tissue, as measured by SDS-PAGE and spectrophotometry respectively (De Vriese et al., 2006). Analysis of peripheral blood mononuclear cells (PBMCs) of patients on LZD treatment for various non-TB related infections, shows a decrease in levels of voltage-dependent anion channel protein (VDAC), a mitochondrial marker. It also decreases cytochrome c oxidase subunit II (COX-II) protein levels, mitochondrial mass and complex IV activity, correlating with an increase in downstream apoptosis (Garrabou et al., 2017). Despite its severe side effects however, this drug is associated with high efficacy in the treatment of MDR-TB, eliciting strong anti-inflammatory effects in human cells in the process (Garcia-Roca et al., 2006). Indeed, LZD inhibits the secretion of IL-6, TNF-α, and IL-1RA in LPS-stimulated PBMCs (Garcia-Roca et al., 2006) and reduces bronchoalveolar lavage (BAL) fluid levels of cytokines including IL-6, IL-1β, Interferon-γ, and IL-17 in a mouse model of MRSA pneumonia (Chen et al., 2013). Additionally, LZD treatment reduces BAL fluid levels of IL-1β, TNF-α, and IL-6 in C57BL/6 mice infected with MRSA BAA-1695, attenuating acute lung injury (Verma et al., 2019).

Bedaquiline (BDQ), an inhibitor of bacterial F1/FO ATP-synthase (Andries et al., 2005), shows differential effects on rat liver mitochondria at opposing concentrations (Belosludtsev et al., 2019). BDQ suppresses the activity of complexes I, II, and III at high concentrations and is suggested to activate complex I at low concentrations in rat liver mitochondria (Belosludtsev et al., 2019). BDQ has also been shown to reduce MMP and increase ROS production in human breast cancer cells, functional effects that significantly alter downstream respiratory profiles in these cells (Fiorillo et al., 2016). Interestingly, a recent study reported that BDQ did not affect MMP or ROS production but still activated host responses including phagosome-lysosome fusion and autophagy which were linked to enhanced intracellular killing in *Mtb* infected hMDMs, even when infected with a BDQ-resistant *Mtb* strain (BDQr-*Mtb*) (Giraud-Gatineau et al., 2020). Therefore, despite having no direct bactericidal effect in these *Mtb*-infected hMDMs, BDQ could still elicit beneficial off-target bactericidal effects in these cells by modulating host function. Thus, the possible effects of TB antimicrobials on mitochondrial function highlights their potential use as a HDT strategy and highlights the importance of examining the effects of these antimicrobials further. Even though the majority of studies examining the effects of TB antimicrobials on mitochondrial alterations have focused on toxicology, many antimicrobials alter host mitochondrial dynamics with beneficial outcomes.

## Tuberculosis Antimicrobials Alter Host Bioenergetics

Various alterations in host immunometabolic profiles occur as a result of *Mtb* infection. These studies have highlighted the important role metabolism, namely glycolysis, oxidative phosphorylation (OXPHOS), and the citric acid (TCA) cycle play in the host response to *Mtb* infection (Gleeson et al., 2016; Cumming et al., 2018; Huang et al., 2018). These changes in host immunometabolic pathways are also dependent on the viability, virulence, and the immunomodulatory potential of *Mtb* (Cumming et al., 2018). Notably, fluctuations in metabolism are also associated with specific macrophage polarization states, which are coupled with the production of various pro-inflammatory cytokines, such as IL-1β and TNF-α (Gleeson et al., 2016; Huang et al., 2018). With growing evidence mounting highlighting the important roles glycolysis and OXPHOS play during *Mtb* infection (Cox et al., 2020; Phelan et al., 2020), examining how antimicrobials affect these metabolic pathways is warranted. Anti-tuberculosis agents that alter host immunometabolism could also help to modulate key inflammatory responses and boost pathogen clearance, thereby improving current TB treatment strategies. Similarly, identifying unwanted effects that these antimicrobials illicit on host bioenergetics could also be clinically relevant.

The first line drug ethambutol (EMB) directly disrupts the ETC, targeting copper chelation and inhibits complex IV activity in human fibroblasts, resulting in a decrease in OXPHOS efficiency, as measured by a reduction in oxygen consumption (Guillet et al., 2010). It is long known that another first line drug, RIF, decreases succinate dehydrogenase (SDH) activity in liver cells of mice infected with *S. aureus*, an effect also observed in uninfected samples (Balakliets et al., 1982; Saksena et al., 1994), indicative that RIF also affects the process of OXPHOS. This reduced activity of SDH may suggest an anti-inflammatory role for RIF as SDH can drive mitochondrial ROS production and induce an inflammatory state in murine macrophages (Mills et al., 2016). This supports the previously mentioned study where RIF decreased IL-1β and TNF-α production in LPS and *Staphylococcus aureus* stimulated monocytes (Ziglam et al., 2004). To compensate for a reduction in SDH activity and in the amount of ATP being produced through oxidative means, RIF may be expected to increase glycolysis as a result, but this was not investigated. However, RIF and LZD, have been found to increase lactate production in primary human osteoblasts (PHOs), indicative of increased glycolytic activity (Duewelhenke et al., 2007). Moreover, as eluded to earlier, since LZD-treatment also decreases complex I, III, IV, and V activity in the muscle and liver of LZD-treated rats, it is likely that this scale of mitochondrial dysfunction significantly affects the mitochondria's ability to undergo the process of OXPHOS (De Vriese et al., 2006). A progressive reduction in oxygen consumption resulting in an increase in blood lactate levels has also been reported in a patient during LZD-induced lactic acidosis, indicative of an increase in glycolysis (Protti et al., 2016). Moreover, in addition to the ability of an antimicrobial to mitigate mitochondrial function, and the inherent connectivity of OXPHOS, glycolysis and the TCA cycle, it is feasible that drug-induced alterations in OXPHOS also modulate the activity of the TCA cycle and glycolysis. For example, if an antimicrobial resulted in a decline in ATP levels due to its ability to negatively affect ATP production through OXPHOS, this could result in the upregulation of glycolysis and increased flux through the TCA cycle, to compensate for the deficiency in ATP levels (Cloonan et al., 2016; Aridgides et al., 2019). Consequently, such increases in glycolysis could also be associated with a more oxidative and pro-inflammatory microenvironment in immune cells (Cloonan et al., 2016; Aridgides et al., 2019). Indeed, LZD-treated THP-1 monocytes stimulated with LPS exhibit increased transcript levels of IL-1β, TNF-α, IL-6, and IL-10 (Bode et al., 2015). Interestingly however, LZD inhibits IL-1β, TNF-α, and IL-6 secretion in mixed PBMCs stimulated with LPS (Garcia-Roca et al., 2006), suggesting that LZD exhibits differential effects on immune cells within mixed PBMC populations, thereby differentially affecting the overall cytokine response.

The drug BDQ disrupts OXPHOS activity, by inhibiting oxygen consumption and ATP production in MCF7 breast cancer cells, decreasing glycolytic activity in the process (Fiorillo et al., 2016). However, in normal human fibroblasts, the opposite is observed with similar BDQ concentrations, wherein BDQ boosts both OXPHOS and glycolytic activity (Fiorillo et al., 2016). In agreement with our hypothesis that antimicrobials have the potential to modulate host immune function, hMDMs treated with BDQ and infected with a BDQ-resistant *Mtb* H37Rv strain, exhibit decreased basal glycolysis and glycolytic capacity (Giraud-Gatineau et al., 2020). OXPHOS or mitochondrial respiration was not affected, however (Giraud-Gatineau et al., 2020). Moreover, in the same cells, BDQ could induce host innate immune resistance, activating autophagy at low concentrations without any severe side effects. Furthermore, hMDMs treated with BDQ also resulted in enhanced killing of BDQ-resistant bacterial species, including *S. Aureus* and *Salmonella typhimurium* (Giraud-Gatineau et al., 2020). This further highlights a protective function for BDQ and provides encouraging evidence for its immunomodulatory effects on host function.

Low concentrations of the antimicrobials moxifloxacin (MXF) and CIP also increase lactate production in primary human cells and cell lines, however, at high concentrations, these drugs have the opposite effect, decreasing glycolytic activity, as measured by reduced extracellular lactate production (Duewelhenke et al., 2007). Furthermore, both drugs exert dose-dependent anti-inflammatory effects on cytokine production. LPS-induced secretion of IL-1β and TNF-α are reduced with MXF and CIF treatment in human THP-1 cells and peripheral blood monocytes, and in rat cortical microglial cells, respectively (Weiss et al., 2004; Zusso et al., 2019). Since increased glycolytic rates are commonly associated with an augmented pro-inflammatory response (Gleeson et al., 2016), antimicrobials that illicit increases in glycolysis and/or pro-inflammatory responses could also be beneficial to the host and aid in early clearance of the infection (Dawson et al., 2009; Phelan et al., 2020). Equally, they could also encompass anti-inflammatory potential which could help to mitigate progressive inflammatory phenotypes. The use of clofazimine (CFZ) is also associated with a shift in host metabolism. CFZ increases lactate:glucose ratios in mice, indicative of increased glycolytic activity (Trexel et al., 2017). Moreover, decreases in urinary excretion of the TCA cycle intermediates succinate and α-ketoglutarate are also observed upon CFZ treatment (Trexel et al., 2017). Such CFZ-induced disruptions in these key metabolic intermediates likely indicates immunomodulatory activity. In fact, CFZ has been shown to reduce inflammatory responses in murine macrophages, increasing circulating IL-1RA and decreasing IL-1β and TNF-α secretion levels (Yoon et al., 2016).

In conclusion, future studies are required in elucidating how TB antimicrobials affect mitochondrial function and bioenergetic profiles *in vitro* and in the lungs of patients with TB. Although limited literature on this topic highlights some contradictory findings across different cell types and gives us some insight into how these antimicrobials can affect these processes, more studies are needed specifically detailing and characterizing these effects in humans, particularly using primary cell models. For example, as human alveolar macrophages are the first line of defense during early TB infection and the metabolic state of these cells underpins their response to infection, initial experiments examining how TB antimicrobials affect glycolytic and OXPHOS profiles, and mitochondrial function, in primary human macrophages during *Mtb* infection, could provide significant insight into this current research gap. Moreover, while taking into the account the resistance status of a *Mtb* strain, administering a TB patient suffering from an adverse inflammatory response with a TB drug that elicits anti-inflammatory properties, instead of a drug known to promote pro-inflammatory or oxidative processes, could aid in the treatment process and offer alternative treatment modalities. Thus, it is not inconceivable that a patient suffering from multiple drug-resistant TB administered with an array of second line drugs is also administered a first line drug known to elicit anti-inflammatory or pro-autophagic properties; the latter first-line drug could help to alleviate the inflammatory burden within the lungs or eradicate *Mtb* infection respectively. In addition to reducing the burden to patients, uncovering additional benefits to these antimicrobials could also help to decrease treatment times, lower costs and inform future studies. These studies should also directly compare these effects across different antimicrobials, drug combinations, drug doses, and immune cell types. Moreover, although first line antimicrobials for the most part are directly ineffective against TB-resistant strains, their immunomodulatory effects on host function could still be therapeutically beneficial even in patients with MDR-TB and XDR-TB. Additionally, due to their ability to directly augment immune function, some of these antimicrobials (antibiotics included), could also help infected immune cells to fight a variety of both bacterial and viral infections.

## Author Contributions

JP: conceptualization and software. JK and JP: funding acquisition and administration. JP and CC: visualization and writing-original draft. All authors: validation, writing-review, and editing. All authors contributed to the article and approved the submitted version.

## Conflict of Interest

The authors declare that the research was conducted in the absence of any commercial or financial relationships that could be construed as a potential conflict of interest.
